# Cost-Effectiveness of Pooled Nucleic Acid Amplification Testing for Acute HIV Infection after Third-Generation HIV Antibody Screening and Rapid Testing in the United States: A Comparison of Three Public Health Settings

**DOI:** 10.1371/journal.pmed.1000342

**Published:** 2010-09-28

**Authors:** Angela B. Hutchinson, Pragna Patel, Stephanie L. Sansom, Paul G. Farnham, Timothy J. Sullivan, Berry Bennett, Peter R. Kerndt, Robert K. Bolan, James D. Heffelfinger, Vimalanand S. Prabhu, Bernard M. Branson

**Affiliations:** 1 Division of HIV/AIDS Prevention, Centers for Disease Control and Prevention, Atlanta, Georgia, United States of America; 2 Wadsworth Center, New York State Department of Health, Albany, New York, United States of America; 3 Retrovirology Section, Florida Bureau of Laboratories, Jacksonville, Florida, United States of America; 4 Sexually Transmitted Disease Program, Los Angeles Department of Health Services, Los Angeles, California, United States of America; 5 LA Gay and Lesbian Center, Los Angeles, California, United States of America; University of Connecticut, United States of America

## Abstract

**Background:**

Detection of acute HIV infection (AHI) with pooled nucleic acid amplification testing (NAAT) following HIV testing is feasible. However, cost-effectiveness analyses to guide policy around AHI screening are lacking; particularly after more sensitive third-generation antibody screening and rapid testing.

**Methods and Findings:**

We conducted a cost-effectiveness analysis of pooled NAAT screening that assessed the prevention benefits of identification and notification of persons with AHI and cases averted compared with repeat antibody testing at different intervals. Effectiveness data were derived from a Centers for Disease Control and Prevention AHI study conducted in three settings: municipal sexually transmitted disease (STD) clinics, a community clinic serving a population of men who have sex with men, and HIV counseling and testing sites. Our analysis included a micro-costing study of NAAT and a mathematical model of HIV transmission. Cost-effectiveness ratios are reported as costs per quality-adjusted life year (QALY) gained in US dollars from the societal perspective. Sensitivity analyses were conducted on key variables, including AHI positivity rates, antibody testing frequency, symptomatic detection of AHI, and costs. Pooled NAAT for AHI screening following annual antibody testing had cost-effectiveness ratios exceeding US$200,000 per QALY gained for the municipal STD clinics and HIV counseling and testing sites and was cost saving for the community clinic. Cost-effectiveness ratios increased substantially if the antibody testing interval decreased to every 6 months and decreased to cost-saving if the testing interval increased to every 5 years. NAAT was cost saving in the community clinic in all situations. Results were particularly sensitive to AHI screening yield.

**Conclusions:**

Pooled NAAT screening for AHI following negative third-generation antibody or rapid tests is not cost-effective at recommended antibody testing intervals for high-risk persons except in very high-incidence settings.

*Please see later in the article for the Editors' Summary*

## Abstract 2

Angela Hutchinson and colleagues conducted a cost-effectiveness analysis of pooled nucleic acid amplification testing following HIV testing and show that it is not cost-effective at recommended antibody testing intervals for high-risk persons except in very high-incidence settings.

## Editors' Summary

**Background:**

Since 1981, acquired immunodeficiency syndrome (AIDS) has killed about 25 million people and about 30 million people are now infected with the human immunodeficiency virus (HIV), which causes AIDS. HIV, which is most often transmitted through unprotected sex with an infected partner or injection drug use, infects and kills immune system cells, leaving infected individuals susceptible to other infectious diseases. The first, often undiagnosed stage of HIV infection—acute HIV infection (AHI)—lasts a few weeks and sometimes involves a flu-like illness. During AHI, the immune system responds to HIV by beginning to make antibodies that recognize the virus but seroconversion—the appearance of detectable amounts of antibody in the blood—takes 6–12 weeks. During the second, symptom-free stage of HIV infection, which can last many years, the virus gradually destroys the immune system so that by the third stage of infection unusual infections (for example, persistent yeast infections) begin to occur. The final stage of infection (AIDS) is characterized by multiple severe infections and by the development of unusual cancers.

**Why Was This Study Done?:**

Antiretroviral drugs control HIV infections but don't cure them. It is very important, therefore, to prevent HIV transmission by avoiding HIV risk behaviors that increase the risk of HIV infection such as having sex without a condom or with many partners. Individuals with AHI in particular need to avoid high-risk behaviors because these people are extremely infectious. However, routine tests for HIV infection that measure antibodies in the blood often give false-negative results in people with AHI because of the time lag between infection and seroconversion. Nucleic acid amplification testing (NAAT), which detects HIV genetic material in the blood, is a more accurate way to diagnose AHI but is expensive. In this study, the researchers investigate whether pooled NAAT screening (specimens are pooled before testing to reduce costs) for AHI in clinic settings after third-generation antibody testing is a cost-effective HIV prevention strategy. That is, does the gain in quality-adjusted life years (QALY, a measure of the quantity and quality of life generated by healthcare interventions) achieved by averting new HIV infections outweigh the costs of pooled NAAT screening?

**What Did the Researchers Do and Find?:**

The researchers combined effectiveness data from a US study in which AHI was detected using pooled NAAT in three settings (sexually transmitted disease [STD] clinics, a community clinic serving men who have sex with men [MSM], and HIV counseling/testing sites) with a “micro-costing” study of NAAT and a mathematical model of HIV transmission. They then calculated the costs per QALY gained (the cost-effectiveness ratio) as a result of HIV prevention by identification and notification of people with AHI through pooled NAAT screening compared with repeat antibody testing. Pooled NAAT for AHI screening following annual antibody testing (the recommended testing interval for high-risk individuals), they estimate, would cost US$372,300 and US$484,400 per QALY gained for the counseling/testing sites and STD clinics, respectively, whereas pooled NAAT for AHI screening was cost-saving for the community clinic serving MSM. The cost-effectiveness ratio increased for the counseling/testing sites and STD clinics when the antibody testing interval was decreased to 6 months but remained cost-saving for the community clinic. With an antibody testing interval of 5 years, pooled NAAT was cost-saving in all three settings.

**What Do These Findings Mean?:**

Cost-effectiveness ratios of US$100,000–US$200,000 are considered acceptable in the US. These results suggest therefore, that the cost of pooled NAAT screening for AHI following negative third-generation antibody testing is not acceptable at the recommended testing interval for high-risk individuals except in settings where HIV infection is very common such as clinics serving MSM. The researchers reach a similar conclusion in a separate cost-effectiveness analysis of pooled NAAT screening following a negative rapid HIV test. Although the accuracy of these results depends on numerous assumptions made in the cost-effectiveness analyses (for example, the degree to which awareness of HIV status affects the behavior of people with AHI), sensitivity analyses (investigations of the effect of altering key assumptions) show that these findings are not greatly affected by changes in many of these assumptions. Thus, the researchers conclude, NAAT screening should be reserved for settings that serve populations in which there are very high levels of new HIV infection.

**Additional Information:**

Please access these Web sites via the online version of this summary at http://dx.doi.org/10.1371/journal.pmed.1000342.

- The US Centers for Disease Control and Prevention provides information on HIV infection and AIDS and on HIV testing and diagnosis
- HIV InSite has information on all aspects of HIV/AIDS
- Information is available from Avert, an international AIDS nonprofit organization on many aspects of HIV/AIDS, including HIV testing (in English and Spanish)
- MedlinePlus has links to further resources on AIDS (in English and Spanish)
- The UK National Institute of Health and Clinical Excellence has a page on measuring effectiveness and cost-effectiveness

## Introduction

Acute HIV infection (AHI) is the stage of disease immediately after HIV acquisition and before HIV antibodies are detectable, when viral replication and shedding peak [1]. Because persons with AHI are highly infectious, are probably unaware of their status, and may still be engaging in high-risk behaviors, identifying persons with AHI offers an important opportunity for HIV prevention. However, the diagnosis of AHI is challenging because it involves expensive laboratory-based nucleic acid testing and modification of current HIV screening algorithms.

To screen for AHI, a nucleic acid amplification test (NAAT) that detects the presence of HIV RNA during the window period before antibodies are detectable is performed on seronegative specimens. Pooled NAAT, in which specimens are pooled before testing, is employed because individual NAAT is prohibitively expensive [2]. Pooled NAAT is routinely used to detect AHI in blood donors [3], and several studies have shown that pooled NAAT after HIV antibody screening is feasible in clinic settings [4]–[9]. However, these studies screened for HIV antibodies with first- or second-generation enzyme immunoassays (EIAs) that are less sensitive for early infection because they detect only immunoglobulin G (IgG). Third-generation EIAs detect IgG and also immunoglobulin M (IgM), the first class of antibody to appear after infection, and thus detect HIV infection earlier than first- or second-generation EIAs, resulting in fewer antibody-negative cases to be detected by NAAT [2],[10]. It is also important to evaluate the use of rapid testing in combination with pooled NAAT, given that rapid tests, though less sensitive than third-generation laboratory-based EIAs during early infection, are increasingly being used for screening, as results are available the same day as testing, which increases numbers of persons who learn their test results [10]–[12].

No study to date has evaluated the cost-effectiveness of conducting pooled NAAT screening for AHI in clinic settings; and cost-effectiveness data are needed to inform policy around AHI screening including an evaluation of the relationship between cost-effectiveness of AHI screening in settings with varying HIV incidences [9],[13],[14]. We conducted a cost-effectiveness analysis of pooled NAAT screening for AHI after antibody testing with both third-generation EIAs and rapid tests in three settings.

## Methods

Our analysis was conducted concurrently with the Centers for Disease Control and Prevention (CDC) multisite AHI study that used pooled NAAT screening for AHI detection in different clinical settings [15]. We constructed an Excel-based model to compare the cost and effectiveness of screening for AHI after HIV antibody screening. Our analysis, which includes a micro-costing study of NAAT testing and a cost-effectiveness model that incorporates the benefits attributable to reduced HIV transmission, conforms to the reference case recommendations of Gold [16]. All costs are reported in 2008 US dollars, future costs are discounted at a 3% annual rate, and outcomes are expressed as cost per quality-adjusted life year (QALY) gained because of cases of HIV infection averted due to pooled NAAT screening. Our incremental cost-effectiveness analysis compares pooled NAAT screening for AHI with screening for HIV antibodies only.

We calculated the total number of persons identified with AHI and the number of potential HIV transmissions averted due to NAAT screening in three settings. We did not include benefits of antiretroviral therapy (ART) to persons identified with AHI, given inconclusive evidence for the effectiveness of ART during AHI. We also assumed that most persons diagnosed with AHI would start ART when their CD4 count declined below 350 cells/µl, and this has been estimated to occur 6–8 y after infection [17]–[20]. QALYs and lifetime medical costs were calculated on the basis of published estimates. We estimated the cost per QALY gained from a societal perspective and the cost per specimen collected and case of HIV identified and notified from the provider perspective.

### Population, Setting, Design

Effectiveness data were obtained from the CDC AHI study that conducted pooled NAAT after third-generation EIA (Genetic Systems 1/2 +O, BioRad Laboratories) in Florida and both a first-generation assay (Vironostika HIV-1 Microelisa, Biomerieux Inc) and third-generation EIA in Los Angeles [15]. We compared the cost and effectiveness of NAAT screening in three settings with different rates of HIV positivity (defined as the number of positive HIV tests/the number of persons tested during the CDC AHI study) and AHI positivity (defined as the number of AHIs identified/the number of persons screened with pooled NAAT during the CDC AHI study): (1) HIV counseling and testing sites (HIV positivity, 1.2%; AHI positivity, 0.01%), (2) municipal sexually transmitted disease (STD) clinics (HIV positivity, 1.0%; AHI positivity, 0.02% ), and (3) a community clinic serving men who have sex with men (MSM) in Los Angeles (California) (HIV positivity, 1.8%; AHI positivity, 0.21%). We used data by Louie et al. [11] to approximate the yield of NAAT after antibody testing with a rapid test (see Text S1). Our analysis is based on pooled NAAT after antibody testing with either a third-generation EIA or a rapid test.

### NAAT Screening Protocol

Pools of 16 EIA-negative specimens were tested with NAAT (Aptima HIV-1 RNA qualitative assay, Gen-Probe Inc). Pools with positive results underwent resolution testing in which all specimens were tested with NAAT individually to identify the positive specimen(s) and, if repeatedly positive, additionally tested with viral load quantification (Versant HIV-1 RNA-branched DNA assay v. 3.0, Siemens Inc.). All persons with EIA-negative/RNA-positive test results were considered presumptive cases of AHI. Experienced disease intervention specialists (DIS) notified presumptive cases of their results and initiated follow-up confirmatory testing with Western blot and partner services to identify potentially infected partners [15].

### NAAT Screening Program Costs: Micro-Costing Study

Program costs were calculated for the addition of pooled NAAT to an existing HIV testing program. To derive labor costs, we conducted time-motion studies in the laboratories that performed NAAT, and the DIS maintained logs of the time, effort, and travel required to notify newly identified AHI cases and conduct partner services. We conducted a detailed valuation of the costs of NAAT, including reagents, equipment, labor, consumables, quality assurance procedures, and shipping costs (incurred because NAAT is typically conducted at high-volume laboratories). Screening costs were calculated as an average cost per specimen tested with NAAT. Costs incurred after presumptive AHI was detected included DIS labor and travel costs and laboratory costs for confirmatory testing, based on the actual number of AHI cases detected in each setting. Table 1 summarizes the cost data; detailed information is provided in Text S1.

**Table 1:** Cost parameters: Expressed as cost per specimen tested unless otherwise noted.

Cost Parameter	Base Case (Sensitivity Analysis) US$	Reference
NAAT laboratory costs		CDC AHI micro-costing study (primary data collection)
Laboratory labor costs initial run	0.73	“
Laboratory shipping labor costs	0.18 (0)	“
Laboratory supervision labor costs	0.20	“
Quality assurance training labor cost for two participants	0.04	“
Reagent cost per test^a^	40 (0–100)	“
Consumables	1.21	“
Shipping variable material costs	0.39 (0)	“
Dedicated laboratory space^b^	0.35	“
**Nonlaboratory costs**		“
Prepare specimens for shipping	0.10 (0)	“
Shipping delivery costs	1.00 (0)	“
**NAAT-positive costs**		“
Resolution labor costs	4.75	“
Resolution reagent cost per test	40 (0)	“
DIS labor costs to notify NAAT + and conduct partner services	66.39	“
DIS mileage^c^	18.57	[44]
**Other cost data:**		
Confirmatory testing (EIA and Western blot)	58.22^d^	[45]
False positive NAAT test costs	286.22^e^	[45],[46]
Lifetime treatment cost of HIV/AIDS	355,867^d^ (177,933–533,800)	[20]

a Reagent costs per tests are based on a reagent rental contract and include reagents, equipment, and training.

b Based on 22 square meters of dedicated lab space at US$37.00 per 0.09 square meter, http://www.colliers.com/Corporate/MarketReports/UnitedStates/.

c Based on 51.07 km at US$0.585 per 1.6 km, http://www.irs.gov/newsroom/article/0,,id=184163,00.html.

d Adjusted to 2008 US dollars.

e Includes, labor, shipping, reagent costs, EIA, and HIV-1 viral load quantification.

### Identification of AHIs with NAAT

Outcome data on the number of AHI cases detected after pooled NAAT in each setting, days to result notification, and proportion receiving NAAT results were taken from the CDC AHI study (Table 2).

**Table 2:** Key effectiveness parameters.

Effectiveness Parameter	Base Case (Sensitivity Analysis)	Source
**AHI positivity; ** ***n*** ** AHIs using third-generation EIA:**		
HIV counseling and testing sites	0.01%, 7	CDC AHI study
Municipal STD clinic	0.02%, 5	CDC AHI study
Community clinic	0.21%, 12	CDC AHI study
**AHI positivity; ** ***n*** ** AHIs w/rapid testing:**		
HIV counseling and testing sites	0.02%, 8.58	Calculated from CDC AHI study [11]
Municipal STD clinic	0.03%, 6.86	Calculated from CDC AHI study [11]
Community clinic	0.44%, 20.58	Calculated from CDC AHI study [11]
**Percent received NAAT results:**		
HIV counseling and testing sites	71%	CDC AHI study
Municipal STD clinic	78% (50%–100%)	CDC AHI study
Community clinic	81%	CDC AHI study
**Mean ** ***n*** ** days after infection to notification of NAAT results:**		
Third-generation EIA	32	Calculated^a^
Rapid testing	48	Calculated^a^
**Median ** ***n*** ** days to notification of +NAAT:**		
HIV counseling and testing sites	15.5	CDC AHI study
Municipal STD clinic	15.5 (7–22)	CDC AHI study
Community clinic	10	CDC AHI study
Transmission rate ratio from persons unaware to aware of HIV serostatus	3.5 (2.6–4.34)	[21]
**Transmission variables:**		
Sexual acute aware daily transmission	0.0005 (0.00095)	Calculated from [21],[47]
Sexual acute unaware daily transmission	0.00195 (0.3306)	[47]
Sexual nonacute aware annual transmission	0.0253 (2.9)	[47]
Sexual nonacute unaware annual transmission	0.0877 (10.1)	[47]
IDU acute aware daily transmission	0.0028	Calculated from [38],[48]
IDU acute unaware daily transmission	0.0036	Calculated from [38],[48]
IDU nonacute aware annual transmission	0.126	[38],[48]
IDU nonacute unaware annual transmission	0.165	[38],[48]

IDU, injection drug user.

a See Text S1.

### HIV Transmissions Averted

We created a mathematical model of HIV transmission from persons with AHI to their partners, on the basis of the awareness of HIV status, stage of disease (acute or nonacute), and behavior (sexual activity or needle-sharing), which is described in detail in Text S1. The model is largely based on the difference in HIV transmissions rates by awareness of HIV serostatus, in which the transmission rate for those unaware of their HIV status is estimated to be 3.5 times that of persons who are aware of their status [21],[22]. The estimated proportions of HIV transmissions from sexual activity and from needle-sharing were based on 2006 national HIV surveillance data [23]. To estimate the potential prevention benefits of NAAT screening, we estimated transmissions averted during the interval from the time a person with AHI learned of their HIV infection until the time that person would have learned of their infection after antibody testing, had NAAT not been used. In the base case, we assumed this interval to be 1 y on the basis of recommendations for annual retesting of persons at high risk. We also used an interval of 6 mo on the basis of HIV screening recommendations for MSM and 5 y as an upper bound on the basis of background testing rates for other populations [24],[25]. In addition, we calculated outcomes for the community clinic that serves a MSM population on the basis of the 3-mo interval recommended for retesting after recent exposure [26]. To estimate the duration of time that NAAT screening would confer awareness of HIV infection compared with antibody screening alone, we calculated the total number of days during acute and nonacute phases of infection that an infected person was aware of his/her infection. We then applied estimated transmission probabilities for each phase to calculate the total number of transmissions averted that were attributable to the NAAT screening program. In sensitivity analysis, we estimated the impact of AHI detection on the basis of symptoms by estimating the proportion of AHI cases that are symptomatic, seek care, and are correctly diagnosed, and removing those estimated symptomatic cases from the AHI cases that could be identified through pooled NAAT screening for AHI [27]–[29]. See Text S1 for details.

### QALYs and Lifetime Medical Costs Attributable to Pooled NAAT Screening for AHI

We estimated QALYs gained from transmissions averted for each setting, antibody testing technology, and timeframe to HIV diagnosis in the absence of pooled NAAT. The QALYs gained were expressed as the difference in QALYs for a partner with and without HIV infection. We used published HIV-related utility weights to estimate QALYs, assuming uninfected persons had a QALY of 1.0 [30]. We assumed 35 y to be the average age at which the partner would have become infected, on the basis of 2006 HIV incidence data, and an additional life expectancy of 32 y for persons infected with HIV [20],[23] and 44.6 y for those who remain uninfected [31]. We used published estimated lifetime HIV treatment costs that were discounted to the time of infection and adjusted them to 2008 US dollars to get a discounted lifetime cost of US$355,867 [20]. Cost-effectiveness estimates were calculated by subtracting projected HIV treatment costs for HIV infections averted from total AHI program costs and dividing by the QALYs gained for each averted HIV infection.

### Sensitivity Analysis

We conducted sensitivity analyses around multiple parameter values including: transmission rates by awareness of HIV serostatus, antibody testing frequency, reagent and equipment costs, shipping costs, receipt of NAAT results, and symptomatic identification of AHI. We also conducted a threshold analysis on AHI positivity rate to determine the AHI positivity at which AHI screening with NAAT becomes cost-effective after third-generation EIA and rapid testing. Our sensitivity analysis was centered on the municipal STD clinic setting because STD clinics have been suggested as places to conduct AHI screening [9],[32]. We also conducted the sensitivity analysis on symptomatic detection of AHI on the community clinic that serves MSM because it is the setting in which NAAT is most likely to be cost-effective.

## Results

Total program costs for NAAT screening after testing using a third-generation EIA during the 22-mo study period were approximately US$458,200 for the counseling and testing sites (54,187 specimens), US$76,900 for the community clinic (5,574 specimens), and US$349,900 for the municipal STD clinics (30,973 specimens) (Table 3). Program costs per specimen tested ranged from US$8.46–US$14.14 in the three settings. The calculated program costs per person identified with and notified of AHI were approximately US$90,000 after screening with a third-generation EIA and US$50,000 after screening with a rapid test for both the counseling and testing sites and municipal STD clinics, and US$7,900 and US$3,800, respectively, for the community clinic.

**Table 3:** Program costs and outcomes of pooled NAAT screening for AHI after third-generation EIA or rapid testing.

Antibody Test	Counseling and Testing Sites		Community Clinic		Municipal STD Clinics	
	3^rd^-Gen EIA	Rapid Test	3^rd^-Gen EIA	Rapid Test	3^rd^-Gen EIA	Rapid Test
Total program costs, US$	458,200	459,000	76,900	79,000	349,900	350,500
Number of specimens tested	54,187	54,192	5,574	5,587	30,973	30,977
Cost per specimen tested	8.46	8.47	13.80	14.14	11.30	11.31
AHI cases identified and notified	5.0	8.44	9.69	20.58	3.89	6.86
Program cost per AHI identified and notified, US$	91,600	54,400	7,900	3,800	90,000	52,000

3^rd^-Gen, third-generation.

### Cost-Effectiveness of AHI Screening after Third-Generation EIA

The addition of pooled NAAT after a negative third-generation EIA to detect AHI had a cost-effectiveness ratio of US$372,300 per QALY gained for the counseling and testing sites, US$484,400 per QALY gained for the STD clinics, and was cost-saving for the community clinic, assuming that the HIV infections detected through NAAT screening would otherwise have been detected by repeat antibody testing 1 y later (base case, Table 4). AHI screening would remain cost-saving in the community clinic, but the cost-effectiveness ratios for the counseling and testing sites and STD clinics would increase to approximately US$1 million per QALY gained if these HIV infections were detected 6 mo later because of more frequent repeat antibody testing. If repeat antibody testing did not occur until 5 y later, the cost per QALY gained was cost-saving in all three settings (Table 4). If HIV infections identified as AHIs were detected by antibody screening within 3 mo of infection because of more frequent retesting at the community clinic that serves MSM population, the cost-effectiveness ratio changed from cost-saving to a positive incremental cost-effectiveness ratio (ICER) of US$5,700 per QALY gained.

**Table 4:** Costs-effectiveness of pooled NAAT screening for AHI after third-generation EIA and rapid testing.

Antibody Test	HIV Counseling and Testing Sites		Community Clinic		Municipal STD Clinics	
	3^rd^-Gen EIA	Rapid Test	3^rd^-Gen EIA	Rapid Test	3^rd^-Gen EIA	Rapid Test
**Base case: HIV diagnosis 1 y after infection w/o NAAT**						
HIV infections averted	0.41	0.51	0.90	1.46	0.32	0.42
HIV treatment costs averted	145,200	181,800	321,700	521,100	113,000	147,800
QALYS gained	2.63	3.29	5.82	9.42	2.04	2.67
Net cost per case averted, US$	977,400	716,300	(236,700)	(467,200)	989,200	695,900
Cost per QALY, US$	372,300	217,900	Cost-saving	Cost-saving	484,400	260,500
**HIV diagnosis 6 mo after infection w/o NAAT**						
HIV infections averted	0.26	0.26	0.60	0.82	0.20	0.21
HIV treatment costs averted	566,800	92,900	1,179,400	293,500	440,900	75,500
QALYS gained	1.67	1.68	3.88	5.30	1.30	1.36
Net cost per case averted, US$	1,669,300	1,665,200	(86,900)	(197,700)	1,657,700	1,651,600
Cost per QALY, US$	997,900	991,700	Cost-saving	Cost-saving	1,274,100	1,154,600
**HIV diagnosis 5 y after infection w/o NAAT**						
HIV infections averted	1.41	2.23	2.95	5.85	1.10	1.81
HIV treatment costs averted	503,600	793,700	1,047,900	2,081,100	391,700	645,200
QALYS gained	9.10	14.35	18.94	37.62	7.08	11.66
Net cost per case averted, US$	(179,800)	(610,900)	(1,021,800)	(2,069,100)	(73,800)	(473,500)
Cost per QALY, US$	Cost-saving	Cost-saving	Cost-saving	Cost-saving	Cost-saving	Cost-saving

Cost-saving denotes a cost per QALY value <0 and is reported as “cost-saving” [16]. Discount rate is 3%.

3^rd^-Gen, third-generation.

### Cost-Effectiveness of NAAT Screening after Rapid Testing

The addition of pooled NAAT for detection of AHI after a negative rapid antibody test had a cost-effectiveness ratio of US$217,900 per QALY gained for the counseling and testing sites, US$260,500 per QALY gained for the STD clinics, and was cost-saving for the community clinic with the base case assumption that HIV infections detected through NAAT screening would otherwise have been detected by repeat antibody testing 1 y later. If HIV infections were detected 6 mo later through repeat antibody testing, AHI screening remained cost-saving in the community clinic, but the cost-effectiveness ratios would be US$991,700 and US$1,154,600 per QALY gained for the counseling and testing sites and municipal STD clinics, respectively. If repeat antibody testing did not occur until 5 y later, pooled NAAT for AHI detection was cost-saving in all three settings (Table 4).

### Sensitivity Analyses

**Figure 1:**
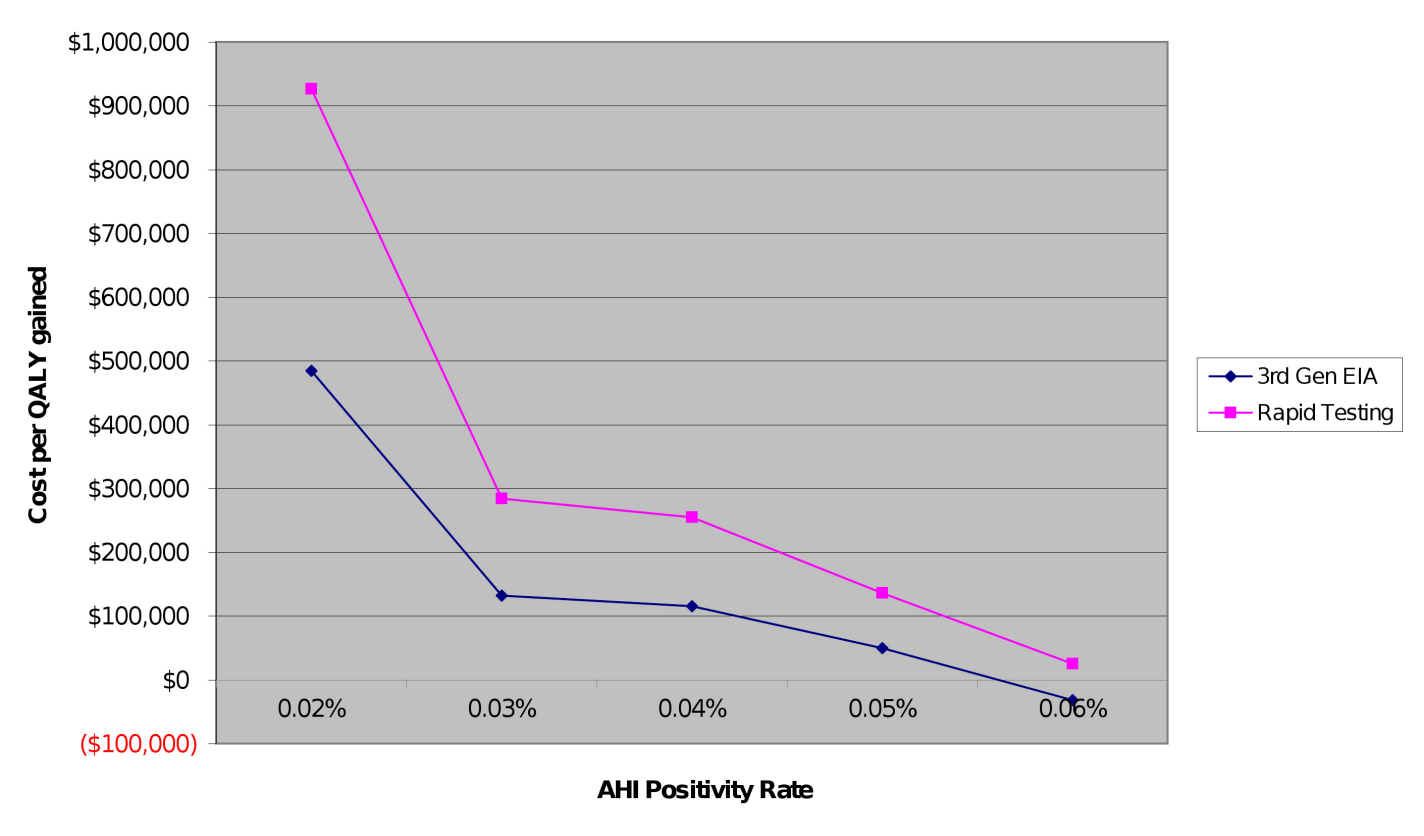
Sensitivity analysis: relationship between AHI positivity rate and cost per QALY gained. Rounded to the nearest tenth of a percentage point. 3^rd^-Gen EIA, third-generation enzyme immunoassay.

The results of the threshold analysis on AHI positivity rate can be found in Figure 1. If AHI positivity is twice as high as observed in the municipal STD clinics (0.04% compared to 0.02%) with NAAT screening after third-generation antibody testing, the cost-effectiveness ratio would fall to US$100,000 per QALY gained. Additionally, for a given AHI positivity rate, cost-effectiveness ratios are higher for pooled NAAT after rapid testing compared to third-generation EIA. At AHI positivity rates of 0.6% or higher, pooled NAAT after third-generation EIA testing becomes cost-saving and cost-effective after rapid testing.

**Figure 2:**
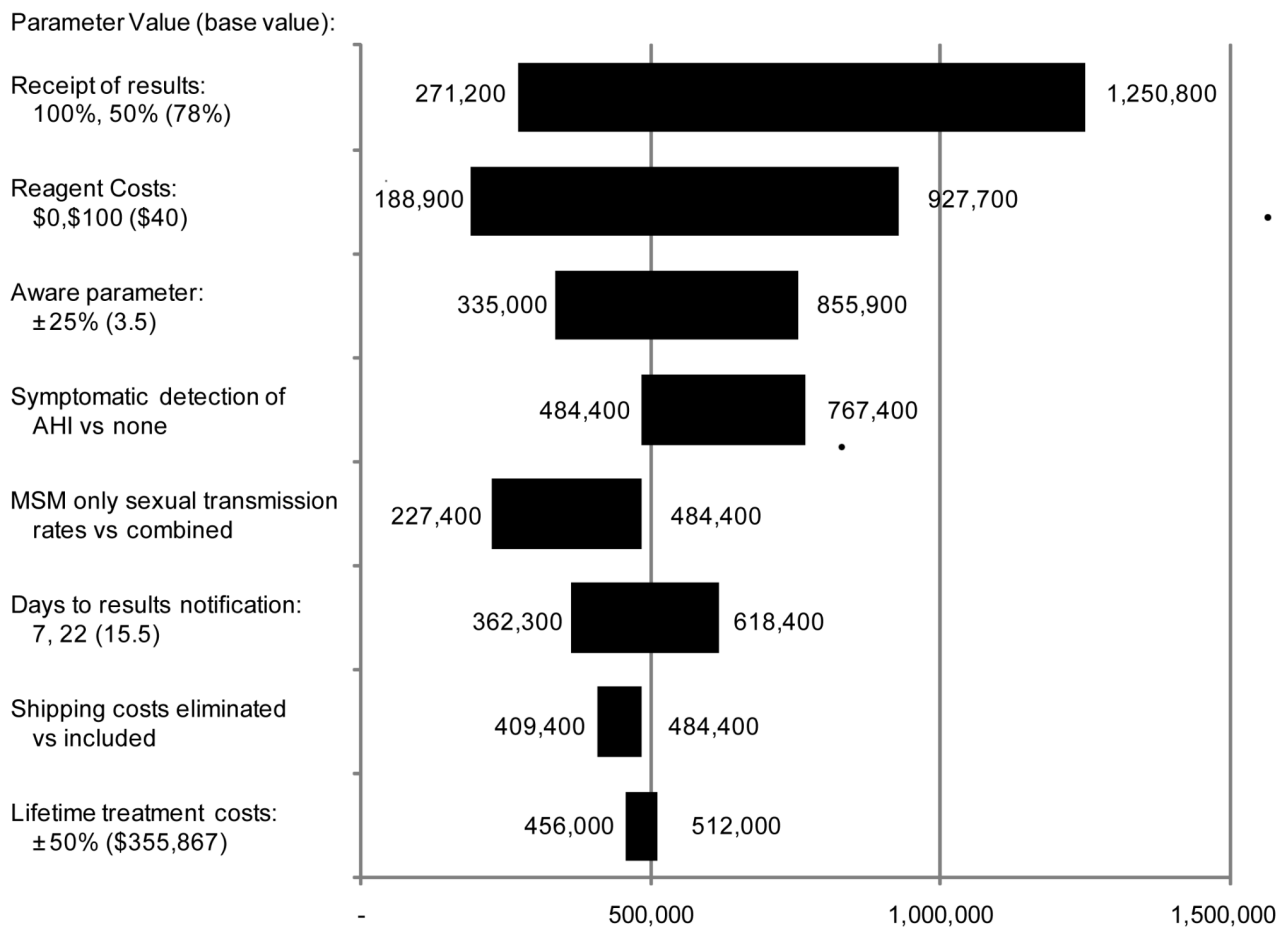
Tornado diagram of sensitivity analysis results. Based on municipal STD clinic data after third-generation EIA assuming a 1-y antibody retesting interval. Base case value  =  US$484,400 per QALY gained.

Figure 2 shows the results of sensitivity analyses for NAAT screening after a negative third-generation EIA for municipal STD clinics under the base case assumption regarding repeat antibody testing at 1 y. Results are not sensitive to a 25% variation in the transmission rate ratio for those unaware compared to those aware of their HIV serostatus for which cost-effectiveness ratios ranged from US$335,000 to US$855,900 per QALY. Results are also not sensitive to use of MSM-only transmission rates versus combined MSM-heterosexual transmission rates, which resulted in cost-effectiveness ratios above US$200,000 per QALY. Cost-effectiveness ratios remained well above US$200,000 per QALY when lifetime medical costs are varied 50%, receipt of results notification is varied from 50% to 100%, and days to results notification are varied from 7 to 22 d. However, in two-way sensitivity analysis, the combination of 100% notification and 7-d receipt of results resulted in a cost-effectiveness ratio of US$73,500 per QALY gained. When we varied reagent cost from US$0.00 to US$100.00, cost-effectiveness ratios ranged from US$188,900 to US$927,700. Cost-effectiveness ratios remained above US$400,000 when shipping costs were eliminated. Finally, accounting for symptomatic detection of AHI increased the cost-effectiveness ratio to over US$700,000 per QALY gained.

Cost-effective ratios remained cost-saving for the community clinic that serves an MSM population with a 25% variation in the transmission rate ratio for those unaware compared to those aware of their HIV serostatus. Additionally, pooled NAAT with symptomatic detection of AHI remained cost-saving for the community clinic (unpublished data).

## Discussion

We found that pooled NAAT screening for AHI after a negative third-generation EIA in clinical settings relevant to public health is not likely to be cost-effective for most settings. Pooled NAAT screening for AHI is cost-effective only when targeted to settings with very high HIV incidence, such as the community clinic, where it remained cost-effective compared with retesting for HIV antibody as often as every 3 mo. At US$370,000–US$1 million per QALY gained in counseling and testing sites and STD clinics, pooled NAAT after a negative third-generation EIA was not within acceptable ranges of cost-effectiveness thresholds of US$100,000–US$200,000 per QALY gained [33],[34] unless high-risk persons were retested very infrequently. When we assessed the use of pooled NAAT to detect AHI after negative rapid HIV tests, which are less sensitive during early infection than third-generation EIAs, the pattern of cost-effectiveness remained the same.

Our findings were most sensitive to AHI positivity rate and assumptions about frequency of antibody testing among infected persons. For the municipal STD clinics to fall within the range of threshold values for cost-effectiveness, the AHI positivity rate would need to nearly double to 0.04% from 0.02%. Our analysis is heavily influenced by the transmission rate ratio of those who are aware versus unaware of their status. However, our findings were not sensitive to a 25% variation in this parameter. When we accounted for symptomatic detection of AHI outside the screening program, AHI screening with pooled NAAT became much less cost-effective for the municipal STD clinics. Thus symptomatic detection of AHI cases can be an important consideration in pooled NAAT for AHI detection in clinical settings. In one AHI screening program, 60% of persons tested were symptomatic and providers suspected AHI in 48% of symptomatic cases [14].

In the base case scenario, we assumed testing at yearly intervals because all settings evaluated in our analysis serve high-risk populations for whom testing is recommended at least annually [24]. However, if retesting is infrequent and HIV infection is not identified until 5 y after infection, the benefits from the infections averted by NAAT screening that accrue over this period make NAAT screening cost-saving in all settings we evaluated. Notably, most of the benefits that make NAAT screening cost-effective during a 5-y interval between retesting occur long after the acute phase, when antibody testing could have been repeated. When we narrowed the testing interval to 6 mo, NAAT was not cost-effective in any setting except the community clinic, and in this setting, it was cost-effective even at a 3-mo testing interval. Recent reports advocate the use of NAAT in high-incidence groups that undergo testing frequently [8]. High testing frequency (e.g., every 6 mo) has been observed among risk groups with the highest incidence of HIV infection, and gay-identified men with AHI typically have been tested in the year before diagnosis [8],[15],[35]. However, we found that the relative benefits of reduced HIV transmission due to NAAT generally diminish as the frequency of antibody testing increases.

The small number of persons with AHI identified by NAAT and the large number of specimens that need to be tested to find them appear to drive our results. Furthermore, approximately 25% of persons with AHI did not receive their test results, and those who did were notified well into the acute phase, on average on days 32 and 48 of the 49-d acute infection period with third-generation EIA screening and rapid testing, respectively. While pooled NAAT detected HIV infection in antibody-negative persons, it did not result in persons learning of their infection early enough to reduce the potential for transmission during the infectious AHI period as much as was expected, which limited the public health benefit of NAAT screening. Timely testing and delivery of NAAT results are essential if the public health benefits of AHI screening are to be optimized and are important considerations for implementing NAAT in real-world settings. If receipt of results dropped to 50% or results notification takes 22 d rather then 15 d, the cost-effectiveness greatly exceeds US$500,000 per QALY. Pooled NAAT screening was cost-effective in sensitivity analysis with 100% notification and 7-d receipt of results. However, it is unclear how feasible 7-d notification of NAAT results is; in another AHI screening program, time to results notification following AHI screening was similar or slightly longer than that reported in the CDC AHI study [14]. Alternate methods for delivering NAAT results such as the internet and telephone might be considered to increase the proportion of persons who receive their NAAT results.

AHI positivity rates in a given population are generally higher with pooled NAAT after rapid testing compared to third-generation EIA screening due to the lower sensitivity of the rapid test during the first few weeks after infection. However, these additional cases might be identified much later in the acute phase because of the longer antibody-negative window period, potentially diminishing some of the reductions in acute phase transmissions, which, in our analysis, made pooled NAAT after rapid testing less cost-effective than pooled NAAT after third-generation EIA screening at similar AHI positivity rates.

Although high infectivity during AHI has been a prime reason to advocate for NAAT screening to detect AHI, our sensitivity analysis suggests that even notifying persons with AHI of their test results within 7 d after screening (which would reduce the interval during which persons with AHI might transmit HIV by 8.5 d) would still result in cost-effectiveness ratios beyond generally accepted threshold values. Larger pool sizes have been suggested as a way to increase AHI screening efficiency and decrease costs. The CDC AHI study was able to retrospectively detect most cases of AHI when retesting samples using a 128-member, two-stage pooling scheme, compared to the original pool size of 16 suggesting an intermediate pool size between 16 and 128 members [36],[37]. It is unlikely, however, that the larger pool size would be more cost-effective because we reduced reagent costs to zero in sensitivity analysis and the cost-effective ratio was still close to US$200,000 per QALY. Additionally, larger pool sizes could increase turn-around time, which will reduce or eliminate the benefit of awareness during the acute phase and may be more costly due to higher reagent costs for resolution testing of larger pools. Smaller pool sizes may decrease turn around time, but reducing time to receipt of NAAT results to 7 d, less than half of the base case value, still resulted in cost-effectiveness ratios in excess of US$350,000 per QALY.

While routine HIV screening has been found to be cost-effective [38], NAAT has been found to be well outside the range of cost-effectiveness at US$7–US$9 million per QALY gained when evaluated in the context of volunteer blood donation screening for HIV [39],[40]. We believe our study to be the first micro-costing study of pooled NAAT in clinical settings. Our analysis includes a detailed valuation of labor costs associated with NAAT screening, and shipping costs, which accounted for 20% of total costs in our study and are likely to play a continuing role in any NAAT screening program because of the need for pooled testing at a regional, high-complexity laboratory.

Our study clearly illustrates that NAAT screening for AHI should be reserved for settings that serve the highest-incidence populations, or circumstances such as donor screening where cost-effectiveness is not a primary consideration [41],[42]. The challenge for public health providers will be to determine how to identify such settings and improve the yield of NAAT screening [9]. HIV positivity and AHI screening yield are not necessarily correlated [15]. Thus, other metrics are needed to identify settings in which AHI screening is likely to be cost-effective. Similar analyses will need to be conducted with alternatives to pooled NAAT such as fourth-generation EIA. Fourth-generation EIA detects both HIV antibody and p24 antigen in a single screening test and can be almost as effective as NAAT in identifying AHI with a potentially shorter turnaround time for results [10],[15].

Our study is subject to several limitations. Our analysis is based on a clinical trial of NAAT conducted in three types of settings with different HIV positivity rates. Caution should be used in applying these results to similar settings because of limited information on the correlation between AHI and HIV positivity rates and the relationship of these factors to the prevalence of risk behavior. Although we assessed the costs of false-positive NAAT results, we did not include any quality-of-life adjustments. However, there were only two false positive NAAT tests out of 90,834 specimens screened in this study [15]. We assumed that awareness of AHI would be associated with the same degree of behavior change as has been estimated for awareness of nonacute HIV infection, but data are not yet available to confirm this assumption. Like many other HIV screening cost-effectiveness analyses, calculation of transmissions averted in our study include only first-generation transmissions, which underestimates the total number of infections attributable to each AHI case [39],[43]. Additionally, our transmission model uses the same transmission rates for heterosexuals and MSM, thus our estimates are slightly conservative with regard to MSM populations, though our findings did not change when MSM only transmission rates were used in sensitivity analysis. We did, however, account for variation in transmission risk by stage of infection, which has not typically been done in other cost-effectiveness analyses of HIV screening. We are also limited by possible changes in costs and effectiveness of ART given that persons with AHI will not be treated for several years. We addressed this in sensitivity analysis using a 50% variation in ART costs, which did not appreciably change the cost-effectiveness ratios. Finally, we did not include benefits related to earlier linkage to care due to pooled NAAT; however, this will need to be reevaluated if treatment becomes recommended during acute and early HIV infection.

### Conclusions

Pooled NAAT screening for AHI after negative third-generation EIA or rapid tests is not cost-effective when antibody testing frequency is at recommended levels for high-risk populations except in very high-incidence settings. When antibody testing frequency is 5 y or greater, pooled NAAT screening is cost-saving; however, most of the benefits achieved occur long after the acute phase and could have been achieved with antibody testing alone.

## Supporting Information

Text S1 Technical appendix.
(0.16 MB DOC)

## Abbreviations

AHI: acute HIV infection
ART: antiretroviral therapy
CDC: Centers for Disease Control and Prevention
DIS: disease intervention specialist
EIA: enzyme immunoassay
MSM: men who have sex with men
NAAT: nucleic acid amplification testing
QALY: quality-adjusted life year
STD: sexually transmitted disease
