# Supplementary material for: Cost-Effectiveness of Pooled Nucleic Acid Amplification Testing for Acute HIV Infection after Third-Generation HIV Antibody Screening and Rapid Testing in the United States: A Comparison of Three Public Health Settings
Source: PLoS Med. 2010 Sep 28;7(9):e1000342. doi: 10.1371/journal.pmed.1000342 (PMC2946951; doi:10.1371/journal.pmed.1000342)
Supplement: Text S1 — Technical appendix. (0.16 MB DOC) [file pmed.1000342.s001.doc]

**TECHNICAL APPENDIX S1**

**Cost-effectiveness of Pooled Nucleic Acid Amplification Testing for** **Acute HIV Infection after Third Generation HIV Antibody Screening and Rapid Testing in the United States:**

**A Comparison of 3 Public Health Settings**

1.0 NAAT Program Costs: Micro-costing Study

NAAT Lab Costs

Time-motion studies were conducted in two public health laboratories that performed NAAT, the New York State Department of Health Wadsworth Center in Albany and the Florida Bureau of Laboratories in Jacksonville. All cost data are reported in 2008 U.S. dollars. A total of 8 complete runs were observed in both sites, including 2 runs to resolve positive pools. Data included time for processing, preparing, aliquoting, and pooling specimens, performing the assay, and recording results. We excluded times not directly related to conducting NAAT such as time to receive and sort specimens (because specimens were also tested with EIA) and technician time during the interval required for incubation because other productive activities could be performed concurrently. Specimens from pools with positive results underwent resolution testing in which all the specimens were tested individually to identify the positive specimen(s). This involved retrieval of the frozen specimens, and completing another NAAT run. We assumed the individual specimens were added to other screening runs, but there were some occasions (e.g., on Fridays) when a special run had to be conducted for individual testing. Each run typically included 16 pools of 16 specimens, 3 individual specimens and 6 controls. Labor costs for the run comprised 6.75 hours at a rate of $27.65 per hour for an average per specimen labor cost of $0.73. Labor costs for resolution testing were based on 2.75 hours at an average cost of $4.75 for each of the 16 specimens tested. Labor costs for the screening runs were taken from the New York State Department of Health Wadsworth Center because they aliquoted specimens and used automated pooling.

We calculated program costs as a cost per specimen tested. Costs for confirmatory testing of AHI cases were calculated separately based on the actual number of AHIs in each setting. For resolution runs, a separate test was used for each specimen. We calculated the average number of specimens tested over a 15-month period (May, 2006- August 2007) for both laboratories and the reagents used for screening, resolution, and controls. We then calculated the ratio of reagents used to specimens tested (.16 for the Los Angeles laboratory and .09 for the Florida laboratory) and applied this ratio to the absolute number of specimens tested for the entire 22-month project period for each setting in the analysis.

NAAT reagent costs were based on a reagent/rental contract using the actual price charged to the public health laboratory of $4000 per 100 reagent tests. The reagent/rental contract included the reagents and some equipment (Gen-Probe-manual, target capture system, 3 water baths, a laminator and software) and training.

Costs of quality assurance include the labor costs for 2 laboratory staff members to attend an on-site training provided by Gen-Probe. Laboratory supervisory costs were based on a 7% annual effort of a laboratory technologist. Wage rates reflect national data on average hourly wages for medical and clinical laboratory technologists ($25.20 per hour) and medical and clinical laboratory technicians ($17.36 per hour)[1]. Employment benefits required for government workers were calculated as employer costs per hour worked ($2.45) and added to the hourly wage rate [1].

We also included the cost of 240 square feet of dedicated laboratory space for the equipment and surrounding “clean” area required for processing NAAT, calculated based on average class A rental price of $37 per square foot per year, including utilities [2].

All specimens that were NAAT-positive on resolution testing incurred an additional cost of $58.22 (adjusted to 2008 dollars) for an EIA and Western blot [3]. We also included costs for resolving false-positive NAAT results including an individual NAAT run (labor, shipping, reagents), EIA and viral load quantification, $161, and 2 hours of clinic labor costs, totaling $286.22 adjusted to 2008 dollars [3,4].

*Non-laboratory NAAT Costs*

Non-laboratory costs included costs for shipping specimens to the public health laboratory and costs for disease intervention specialists (DIS) to locate and notify persons infected with AHI of their test results. We did not include costs for offering NAAT, pre-test counseling or phlebotomy because clients signed a universal HIV testing consent obviating the need for obtaining a separate NAAT-specific consent form. Costs for notifying NAAT-positive patients of their results were based on time logs completed by the DIS who conducted notification and partner services. These data were collected for 26 persons with AHI identified during the study, 18 of whom accepted partner services. There were 45 named partners and 14 (31%) of those partners were tested. The DIS spent an average of 3.15 hours to notify clients and conduct partner services, based on an average of 4.3 phone calls, 1.9 field visits and 1.3 letters sent. Wage rates of $18.56 per hour were based on national data on average hourly wages for community and social service specialists [1] and $2.45 per hour for government benefit costs [1]. DIS costs also include $0.585 per mile for an average of 31.7 miles traveled per client [5]

*Shipping Costs and Consumables*

A detailed assessment of shipping costs was conducted in July 2007 by the New York State Department of Health public health laboratory. Shipping costs are expressed as an average cost per specimen. Shipping costs were incurred by both the laboratories and the clinics because specimens were shipped from the clinics to the laboratories and shipping materials were returned to the clinics from the laboratories. These costs included labor and materials and shipping carrier costs. Shipping labor costs were calculated based on average annual wages for a laboratory technician plus benefits [1]. The average shipping labor cost per specimen was $0.18 ($0.17 for New York sites and $0.19 for Los Angeles sites) for the laboratory and $0.10 for the clinic. The average cost per specimen for shipping materials was $0.39 and included costs for: insulated shipping container (n=40, $1244); outer corrugated packing (n=40, $87); gel packs (n=97, $826); mailing tape (n=60, $162); specimen bags, absorbent, thermometer, data loggers (n=10, $690); and software to monitor data loggers (n=1, $21.62 amortized over 5 years). Carrier costs, tracked for the month of July for clinics in New York City and Los Angeles, averaged $1.00 per specimen. The average cost of consumables per specimen was $1.21 and included PPT tubes, labels, specimen stoppers, deep well plates, pipette tips, specimen tube stoppers, gloves, and bleach.

NAAT false positive costs consist of labor, shipping and reagent costs for one resolution run, costs for an EIA and viral load and clinic labor costs for 2 hours ([3],[4]).

2.0 Yield of Pooled NAAT after Rapid Antibody Testing

To estimate the number of AHIs that would have been detected among persons screened with rapid HIV tests, we approximated the sensitivity and yield of NAAT after rapid HIV testing using data that compared the relative sensitivity of EIAs and rapid tests on a panel of specimens of acutely infected persons [6]. Of 42 RNA-positive specimens that were non-reactive on the Vironostika HIV-1 Microelisa, 98% were non-reactive with two point-of care rapid HIV tests but only 68% were non-reactive with the third-generation Genetic Systems HIV-1/2 +O EIA [6]. Data from the CDC AHI study sites in Los Angeles that were screened with the Vironostika assay were adjusted using the 0.98 factor to approximate the yield of NAAT screening after rapid antibody tests. Because the Florida study sites screened only with a third-generation EIA, we first estimated the AHI yield after screening with the Vironostika assay and then used the same correction to approximate the yield of NAAT after rapid testing.

1. Calculation of HIV Transmissions Averted Attributable to Pooled NAAT Screening for AHI

We created a mathematical model of HIV transmission from persons identified with AHI to their partners that incorporated effects of awareness of HIV status, stage of disease (acute versus non-acute) and behavior (high-risk-sex versus needle-sharing) represented by formula (1). We modeled transmissions averted as the difference between the transmissions likely to occur during the acute phase by those who were unaware of their infection (acute unaware) and the transmissions likely to occur during the acute phase by those who became aware of their infection (acute aware) because of NAAT screening. We also modeled the difference between the estimated number of transmissions that would have occurred during the non-acute phase from those who were unaware of their infection (non-acute unaware) and those who became aware of their infection earlier (non-acute aware) because of NAAT screening. The proportions of HIV transmissions from risky-sex and from needle-sharing based on national HIV surveillance data were used to assign transmissions averted for the HIV CT site and STD clinic. We assumed 99% of transmissions averted at the community clinic were sexual transmissions [7].

Total transmissions averted = (ΔT_S_* P_S_ + ΔT_IDU_ * P_IDU_)* N (1)

Where,

ΔT_S_ = Sexual transmissions averted per AHI diagnosed and notified

ΔT_IDU_ = IDU transmissions averted per AHI diagnosed and notified

P_S_= proportion of sexual transmissions in the U.S. in 2006 (.865)

P_IDU_= proportion of IDU transmissions in the U.S. in 2006 (.134)

N = Number of AHI diagnosed and notified

Sexual transmissions averted when NAAT is used,

ΔT_S_ = T_noNAAT_ – T_NAAT (2)_

= D_A_ * (μ_AU_  – μ_A A_) + D_NA_ * (μ_NU_  – μ_NA_)

Where,

T_noNAAT_ = Number of transmission without NAAT testing/ AHI diagnosed and notified

T_NAAT_ = Number of transmissions with NAAT testing/ AHI diagnosed and notified

D_A_ = Number of days in the acute phase that the AHI were aware of their serostatus when tested with

NAAT[[1]](#footnote-2)

μ_AU_  = Daily sexual transmission rates for acute unaware persons with AHI (.00195)

μ_AA_ = Daily sexual transmission rates for acute aware persons with AHI (.0005)

D_NA_ = Duration since infection in the non-acute phase when individuals would be diagnosed in the absence of NAAT (1 year, 6 months, and 5 years)

μ_NU_  = Annual sexual transmission rates for non-acute unaware persons with AHI (.0877)

μ_NA_  = Annual sexual transmission rates for non-acute aware persons with AHI (.0253)

IDU transmissions averted when NAAT is used,

ΔT_IDU_ = T_noNAAT_ – T_NAAT (3)_

= D_A_ * (μ_AU_  – μ_A A_) + D_NA_ * (μ_NU_  – μ_NA_)

Where,

T_noNAAT_ = Transmission without NAAT testing/ AHI diagnosed and notified

T_NAAT_ = Transmissions with NAAT testing/ AHI diagnosed and notified

D_A_ = Number of days in the acute phase that the AHI were aware of their serostatus attributable to NAAT testing^1^

μ_AU_  = IDU daily transmission rates for acute unaware persons with AHI (.0036)

μ_AA_ = IDU daily transmission rates for acute aware persons with AHI (.0028)

D_NA_ = Duration since infection in the non-acute phase when individuals would be diagnosed in the absence of NAAT

μ_NU_  = IDU annual transmission rates for non-acute unaware persons with AHI (.165)

μ_NA_  = IDU annual transmission rates for non-acute aware persons with AHI (.126)

To estimate the prevention benefits of NAAT, we calculated the extra interval of time that a NAAT test would permit persons to be aware of their HIV infection compared to an antibody test alone. We first estimated the average length of time from day of infection to day of NAAT testing. Because all AHIs identified through pooled NAAT were antibody-negative, we know that the day of NAAT testing falls during the period between NAAT positivity and antibody positivity, and we assumed that it was equally likely persons with AHI were tested on any given day during that period. The expected value or average would be the mid-point of that period. For example, HIV is detectable with NAAT on day 11 and 3^rd^ generation antibody test on day 22, so we assumed a 3^rd^ generation antibody-negative, NAAT-positive person was tested on day 16.5 after infection [8]. For rapid testing/first generation EIA, HIV is detectable on day 54, so we assumed a rapid antibody-negative, NAAT-positive person was tested on day 32.5 after infection [6,8].

We then determined the average length of time it took persons with AHI to become aware of their status by using the median number of days to notification based on CDC AHI study data: 10 days for the community clinic and a mean of 15.5 days for the HIV counseling and testing sites (17 days) and municipal STD clinics (14 days). We added the estimated number of days from infection that the person was tested using third-generation EIAs (16.5) and rapid testing (32.5) to the mean number of days to notification. We subtracted that number from 49 days, representing the average 49-day acute infection period [9,10,11], to derive the number of days the AHI was aware of their status during the acute period for third-generation EIA (17) and rapid testing (1) for which we applied acute phase transmission rates. Because the prevention benefits of NAAT testing extend beyond the acute phase, we estimated the average length of time individuals identified with AHI through NAAT knew their HIV diagnosis during the non-acute disease stage compared with when they otherwise would have learned. We assumed that these individuals otherwise would have been tested for HIV 1 year later in the base case [12], and 3 months [13], 6 months [14], and 5 years later in sensitivity analyses.

To estimate sexual HIV transmissions averted attributable to the NAAT screening program, we applied a daily sexual transmission rate calculated as the difference between *acute* aware and *acute* unaware transmission and an annual transmission rate calculated as the difference between *non-acute* aware and *non-acute* unaware transmission. This difference represents transmissions averted attributable to AHI screening. Daily and annual sexual transmission rates were based on Pinkerton and updated by Prabhu [15,16](Table 2). These estimates were calculated using the transmission risk ratio (3.5), which represents the ratio of transmissions by persons unaware to aware of their HIV status by Marks et al. [17]. The Marks et al., estimate is based on meta-analytic data that showed a 57% (the lower bound) relative reduction in unprotected sex acts with at risk partners [18]. Because Pinkerton and Prabhu did not report a sexual acute aware transmission rate, we adjusted the sexual unaware transmission rate by the transmission rate ratio. In sensitivity analysis we varied the transmission rate ratio by 25% resulting in a transmission rate ratio of 2.60 as a lower bound and 4.34 as an upper bound. We used these ratios to recalculate the daily and annual transmission rates according to Pinkerton and Prabhu and calculate sexual acute aware transmission rates. The daily and annual sexual transmission rate was applied to both heterosexuals and MSM. Pinkerton and Prabhu calculated sexual transmission rates using an average of male-to-female [9] and male-to-male per-act transmission probabilities [19] . We used these combined transmission rates in the base case because AHI occurred in both at risk groups. In sensitivity analysis, we used the male-to-male per-act transmission probabilities to recalculate the sexual transmission rates for MSM

We used the same method to estimate IDU transmission rates attributable to the NAAT testing program. IDU non-acute transmission rates were based on Sanders [20] and Zaric [21]. IDU acute transmission rates were calculated using the proportionate difference in sexual acute transmission and sexual non-acute transmission (Table 2).

To account for symptomatic detection of AHI cases in sensitivity analysis, we estimate the cumulative proportion of cases that were symptomatic (.67), sought care (.94), and were correctly diagnosed with AHI (.25) [22,23,24] which resulted in 19% of AHI cases that may have been detected due to symptoms. We subtracted that proportion of cases from the AHI cases that were available to be identified due to pooled NAAT screening.

REFERENCES

1. Sample calculation for D_a_

   For 3^rd^ generation EIA, D_a_ = T_A_ - [(TDx_3GEN + TDx_NAAT) / 2 + T_NOTIFY_]=(49 - [(22 + 11) / 2 + 15.5] = 49 - [16.5 + 15.5] = 49 - 32 = 17 , where

   T_A_ = Duration of Acute phase = 49 days

   TDx_NAAT = Day that AHI is detectable with NAAT = 11

   TDx_3GEN = Day that AHI is detectable with 3^rd^ generation EIA = 22

   T_NOTIFY_ = Days to notification = 15.5 [↑](#footnote-ref-2)
